# Picosecond dissipative soliton generation from an ytterbium-doped fiber laser based on a BP/SnSe_2_-PVA mixture saturable absorber

**DOI:** 10.1007/s12200-023-00074-3

**Published:** 2023-07-19

**Authors:** Yuting Ouyang, Jiayu Zhang, Wanggen Sun, Mengxiao Li, Tao Chen, Haikun Zhang, Wenjing Tang, Wei Xia

**Affiliations:** 1 School of Physics and Technology, University of Jinan, Jinan, 250022 China; 2 Shandong Huaguang Optoelectronics Co. Ltd., Jinan, 250101 China

**Keywords:** Dissipative soliton, Ytterbium-doped fiber laser, Mixture of BP/SnSe_2_-PVA, Mode-locking

## Abstract

**Abstract:**

Stable picosecond dissipative soliton pulses were observed in an ytterbium-doped fiber laser employing a high-quality mixture of BP/SnSe_2_-PVA saturable absorber (SA). The modulation depth, saturation intensity, and non-saturable loss of the mixture of BP/SnSe_2_-PVA SA were measured with values of 5.98%, 18.37 MW/cm^2^, and 33%, respectively. Within the pump power range of 150–270 mW, stable dissipative soliton pulses were obtained with an output power of 1.68–4 mW. When the minimum pulse duration is 1.28 ps, a repetition rate of 0.903 MHz, center wavelength of 1064.38 nm and 3 dB bandwidth of 2 nm were obtained. The maximum pulse energy of 4.43 nJ and the signal-to-noise ratio up to 72 dB were achieved at pump power of 270 mW. The results suggest that the BP/SnSe_2_-PVA mixture SA has outstanding nonlinear saturable absorption characteristics and broad ultrafast laser applications.

**Graphical Abstract:**

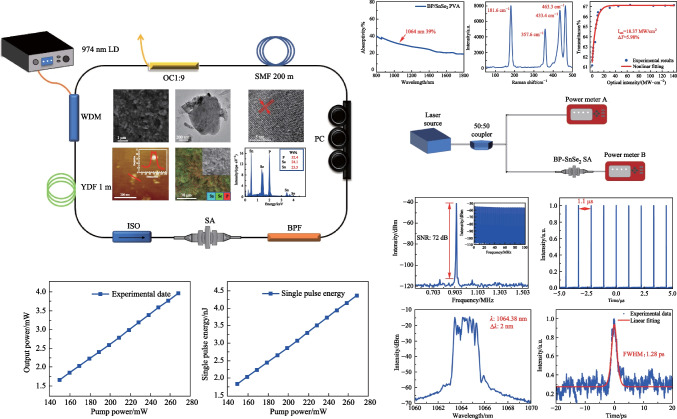

## Introduction

Mode-locked ytterbium-doped fiber lasers have been utilized in a variety of applications in laser processing of material due to their high efficiency, wide gain bandwidth, and compact structure [1–3]. The control of nonlinearity and dispersion in the resonant cavity is primarily responsible for the energy scaling of fiber lasers. Due to their ability to operate in the positive dispersion region, ytterbium-doped fiber lasers (YDFL) can serve as a platform for research into generation of dissipative solitons and associated dynamics [4, 5]. For most mode-locked fiber lasers, dissipative solitons have a much wider pulse width and a lower peak power than conventional solitons and these characteristics make dissipative solitons effectively avoid pulse splitting and significantly improve the single pulse energy. In the normal dispersion region of YDFL, the balance of dissipative soliton interactions can be achieved by introducing bandpass filtering [6–9]. Recently, dissipative solitons have been the subject of numerous investigations. It has been well demonstrated that passive mode locking based on two-dimensional (2D) materials is one of the main methods of generating dissipative solitons. Lu et al. utilized evanescent field interaction with gold nanorods (GNRs), and produced stable dissipative solitons. With pump power of 220 mW at 1041 nm, dissipative soliton pulses with pulse width of 162.3 ps and repetition frequency of 6.649 MHz were successfully produced using GNRs. Signal-to-noise ratio (SNR) up to 77 dB was attained [7]. Chen et al. produced dissipative solitons in an YDFL that was modulated by introducing a mechanically exfoliated NbSe_2_ film as the saturable absorber (SA). The system could deliver stable dissipative solitons with pulse duration of 174 ps, repetition rate of 14.7 MHz, and SNR of 59.8 dB at pump power of 300 mW. These excellent experimental results demonstrate that the 2D material indeed has a good ultrafast nonlinear optical response and has great potential for the study of dissipative solitons [10].

In recent years, many 2D nanomaterials have been successfully used as SAs in passively mode-locked fiber lasers for their advantages of excellent energy conversion efficiency, broadband saturable absorption, and low material cost. Among these 2D materials, the most mature ones are black phosphorus (BP) and transition metal dichalcogenides (TMDs), but single BP and TMDs have poor stability while combining the two materials into a heterostructure have more stable properties [8, 11–14]. Heterostructure generally consists of two or more different materials (mainly 2D materials), which can be combined and stacked together by Van Der Waals (VDW) forces to form new and more complex layer structures [15, 16]. The resulting composites exhibit new physical properties and can be further optimized in terms of optoelectronic properties, thus favoring generation of better mode-locked signals. The materials have also been shown to maintain their optical properties while allowing for electron migration and inter-band jumping through the coupling between the layers. As SAs, heterostructure can widen the applications of 2D materials in nonlinear optics and ultrafast photonics.

In the family of TMDs, SnSe_2_ has appropriate photoelectric properties as a 2D layer material with a tiny band gap; these properties include a reasonable modulation depth, a broad modulation wavelength range, and a tunable band gap, providing a very promising layered structure for SAs [17, 18]. However, as is typical of TMDs, the exciton decay time is long and the pulse width cannot be effectively compressed [19]. According to earlier research works, the heterostructure can successfully enhance the performance of mode-locking, leading to a significantly reduced pulse width and considerably improved pulse stability. In 2019, Liu et al. used a MoS_2_-WS_2_ heterojunction as a SA in an erbium-doped fiber laser and successfully obtained a stable mode-locked pulses with a small pulse duration of 154 fs [20]. In 2017, Liu et al. prepared a graphene/BP heterojunction and used it to produce a mode-locked pulse in an erbium-doped fiber laser. They obtained stable pulses with a pulse duration of 148 fs, which is substantially shorter than that of mode-locked lasers based on single BP [21]. The mixture of BP/SnSe_2_-PVA can be also used as SA. To our best knowledge, there are no studies on the application of this material in modulating Yb-doped dissipative soliton lasers.

In this paper, we have successfully prepared a high-quality SA composed of a mixture of BP, SnSe_2_, and PVA. The BP/SnSe_2_-PVA SA was prepared by the liquid phase exfoliation (LPE) method, and we have characterized the material following the reported works [21–23]. The BP/SnSe_2_-PVA mixture SA was measured to have a good transmittance of 39% at 1064 nm. The saturation intensity, modulation depth and non-saturable loss were 18.37 MW/cm^2^, 5.98%, and 33%, respectively. When the pump power was in the range of 150–270 mW, dissipative soliton pulses were obtained with pulse width of 1.28 ps, repetition frequency of 0.903 MHz, center wavelength of 1064.38 nm, and 3 dB bandwidth of 2 nm, the corresponding output power was in range of 1.68–4 mW. A dissipative soliton mode-locked YDFL was successfully realized in the experiment for the first time.

## Material preparation and characterization

The LPE method, which was adopted due to its convenience of operation and simplicity, was used to prepare the BP/SnSe_2_ PVA film [24–28]. First, 300 mg of BP and SnSe_2_ powders were thoroughly ground in a grinding bowl, then a commixture was made with 10 mL of 30% anhydrous ethanol and sonicated for 24 h. The upper clear layer was taken and mixed with 4 wt% polyvinyl alcohol (PVA) solution at a 1:1 volume ratio. After shaking well to form a homogeneous mixture, 100 μL of BP/SnSe_2_ PVA dispersion was applied to the sapphire substrate with a pipette gun and air-dried in clean room conditions for 24 h. The final BP/SnSe_2_-PVA mixture SA was then obtained.

**Fig. 1:**
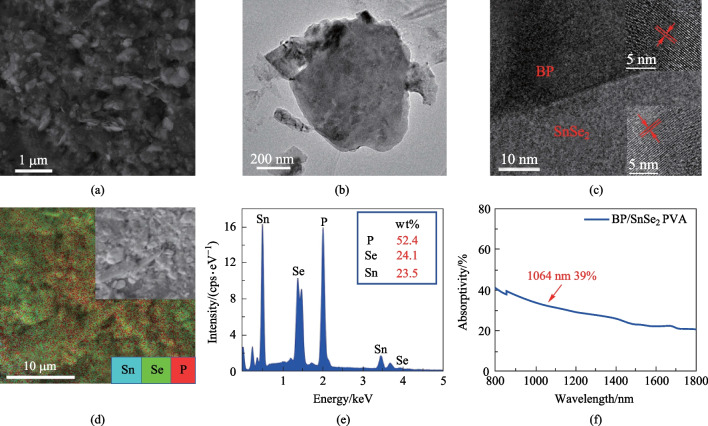
**a** SEM image, **b** TEM mapping image, **c** HRTEM image **d** EDS image, **e** element content analysis map, and **f** absorption spectrum of the BP/SnSe_2_-PVA mixture film

The properties of the BP/SnSe_2_-PVA mixture film were investigated in various ways. First, scanning electron microscopy (SEM) was used to examine the microscopic morphology of the material [14, 29]. Figure 1a shows the SEM image of the BP/SnSe_2_-PVA mixture film; it can be clearly seen that the material has an obvious layered structure. To further investigate the microstructure of the BP/SnSe_2_-PVA mixture films, the transmission electron microscope (TEM) image was also obtained and is exhibited in Fig. 1b. Figure 1c shows the high-resolution transmission electron microscopy (HRTEM) image, exhibiting the lattice structures of BP and SnSe_2_ and their borderline; the inset clearly shows the lattice of BP and SnSe_2_. From the HRTEM image, the BP/SnSe_2_-PVA mixture was further confirmed. Figure 1d shows the energy dispersion spectrum (EDS) that was used for qualitative assessment of material composition. Figure 1d lists that the elements Sn, Se, and P were uniformly distributed, indicating that BP and SnSe_2_ were in full contact. Figure 1e is the element content analysis map, which further indicates the composition of the material. The linear optical response of the BP/SnSe_2_-PVA mixture film was also investigated in our work. The absorption spectrum was measured by UV–VIS-IR spectroscopy and is shown in Fig. 1f; a relatively flat absorption curve between 800 and 1800 nm and a linear absorption coefficient of 39% at 1064 nm were obtained [30–32]. The broadband absorption characteristics of the BP/SnSe_2_-PVA mixture were then determined.

**Fig. 2:**
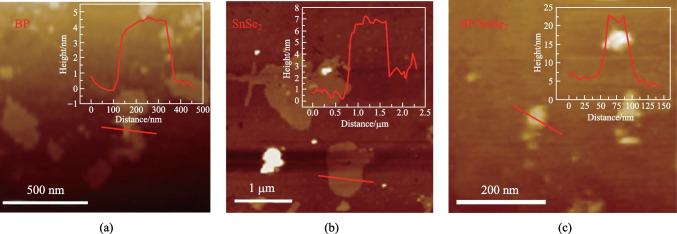
**a** AFM image of BP, **b** AFM image of SnSe_2_, and **c** AFM image of the BP/SnSe_2_-PVA mixture film

**Fig. 3:**
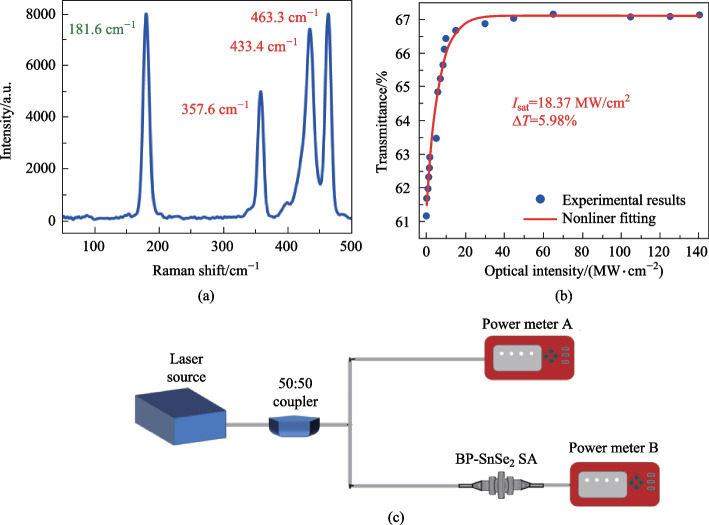
**a** Raman spectrum, **b** measured nonlinear transmission curves of the BP/SnSe_2_-PVA mixture film. **c** Experimental setup of the twin-detector measurement

The as-grown BP/SnSe_2_-PVA mixture was also characterized by an atomic force microscope (AFM). Figure 2a and b show the height and phase pictures of BP and SnSe_2_, respectively, and Fig. 2c shows the AFM map of the BP/SnSe_2_-PVA mixture. Figure 3a presents the Raman spectrum of the samples prepared as described above with a 532 nm laser. The spectrum shows four Raman peaks, located at 357.6, 433.4, and 463.3 cm^−1^ for BP (red labels), and 181.6 cm^−1^ for SnSe_2_ (green label) [33, 34].

To facilitate the integration of SA for mode locked fiber lasers, the BP/SnSe_2_-PVA mixture film was transferred to the end face end of an Yb-doped fiber. The nonlinear optical response of the BP/SnSe_2_-PVA mixture thin-film was measured using the twin-detector measurement method; the measuring setup is shown in Fig. 3c. The transmittance of the material was calculated by the ratio of reference power to signal power, showing the saturated absorption of the BP/SnSe_2_-PVA mixture film. The relevant data of the light sources used for the tests are as follows: central wavelength of 1067 nm, pulse width of 860 fs and repetition frequency of 23.2 MHz.

The nonlinear transmittance curves were fitted using Eq. (1).

1 $$T\left(I\right)=1-\Delta T\times \mathrm{exp}\left(-\frac{I}{{I}_{\mathrm{sat}}}\right)-{A}_{\mathrm{ns}},$$

where *T*(*I*) is transmittance, Δ*T* indicates modulation depth, *I* and *I*_sat_ are the input intensity from the laser and the saturation intensity, respectively, and *A*_ns_ expresses non-saturable absorption loss. The optical saturable absorption response of the BP/SnSe_2_-PVA mixture film was investigated as shown in Fig. 3b, using a twin-detector measurement system. The blue dots in Fig. 3b represents the experimental data. The red curve depicts the fitting curve of the experimental data, indicating the presence of saturable absorption characteristics. From Fig. 3b, the modulation depth, saturable intensity, and non-saturable loss were determined to be 5.98%, 18.37 MW/cm^2^, and 33%, respectively, demonstrating that the BP/SnSe_2_-PVA mixture film is a promising candidate as a SA [35]. In nonlinear transmittance measurement, it was found that the thicknesses of film or wt% ratio of PVA affected the modulation depth and the non-saturable loss of SA. As the thickness of the film increased or the wt% ratio of PVA increased, the modulation depth decreased and the non-saturable loss increased, which is similar to the results of previous studies [36].

## Experiment setup

**Fig. 4:**
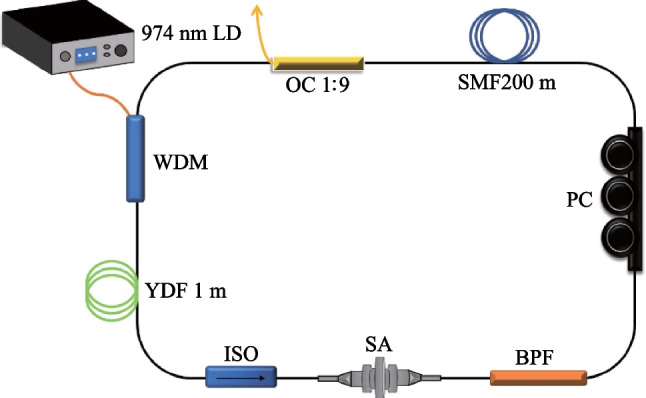
Experimental setup of the mode-locked YDFL

The experimental setup of the mode-locked YDFL based on BP/SnSe_2_-PVA mixture SA is shown in Fig. 4. It consists of a laser diode (LD), a wavelength-division-multiplexer (WDM), an Yb-doped fiber, an optical isolator (ISO), a set of polarization controller (PC), a band pass filter (BPF), and an optical coupler (OC, 1:9). The LD with the output center wavelength of 976 nm. A piece of 1 m Yb-doped fiber (Yb1200-4/125) with peak core absorption of 280 dB/m at 920 nm, group velocity dispersion (GVD) of 24.22 ps^2^/km, numerical aperture (NA) of 0.21, and cladding diameter of 125 μm was applied as the gain medium. A 200 m-long single-mode fiber (SMF) with a GVD of 17.7 ps^2^/km was employed to adapt the intracavity dispersion [37]. The total length of the cavity was 227 m. The ISO was used to maintain the unidirectional transmission of light. The polarization state of light waves in the cavity could be controlled by adjusting the PC. The BPF, operating at 1064 nm with a pass bandwidth of 5 nm, was used to suppress noise signals and mode competition effects. The 1:9 OC was used to output 10% of the laser power to monitor the pulse performance of this YDFL [38, 39]. The pulse properties were analyzed by using an optical spectrum analyzer (Anritsu MS9710C), a power meter (Thorlabs PM100A), and a 1 GHz mixed domain oscilloscope (Tektronix MDO3102).

## Results and discussion

In our experiments, in order to be able to state more precisely that the production of dissipative solitons is entirely due to the presence of the BP/SnSe_2_ mixture SA, we performed a comparison experiment without BP/SnSe_2_ mixture SA under the equivalent conditions. In this experiment, even with careful adjustment of the pump power over the entire range, no mode-locked output pulses were observed. The results clearly confirm that the dissipative soliton was achieved solely due to the nonlinear effects exerted by the SA. The output properties of the YDFL with the BP/SnSe_2_ SA was investigated in detail. To ensure the reliability of the experimental results, we tested the threshold of the laser and achieved an output power of 0.119 mW at threshold pump power of 70 mW. The threshold pump power is quite low compared to that reported in related literatures [20, 21], which demonstrated the low loss of the overall cavity.

**Fig. 5:**
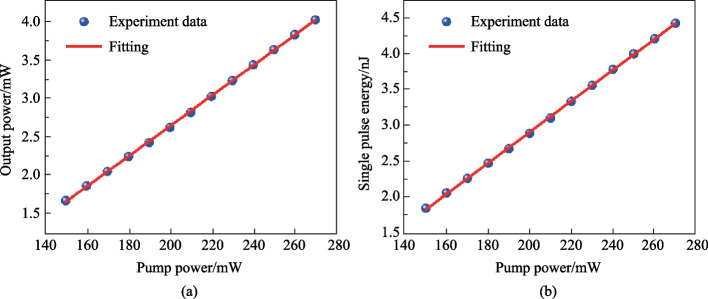
**a** Output power and **b** single pulse energy under different pump powers for the YDFL with the BP/SnSe_2_ SA working in dissipative soliton mode-locking state

With the pump power increased to 140 mW, dissipative soliton mode-locking pulses were observed. The mode-locking state was achieved in the pump power range of 140–250 mW, and the mode-locking state began to become unstable when the pump power exceeded 250 mW. Figure 5a shows the relationship between pump power and output power in the pump power range of 140–250 mW, corresponding to a slope efficiency of 1.5% by detailed calculation. Figure 5b depicts the relationship between pump power and single pulse energy, both showing a clear upward trend, indicating the stable operation of this YDFL.

**Fig. 6:**
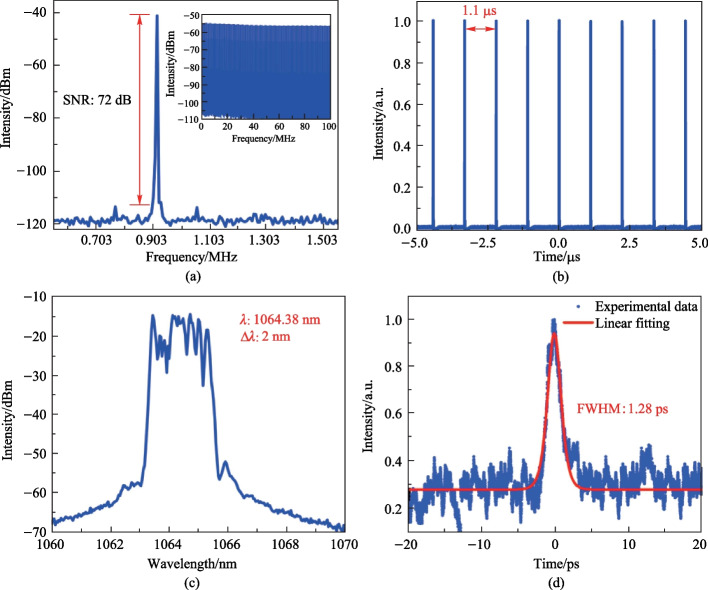
**a** Radio frequency spectrum of the soliton. The inset shows the wide-band RF spectrum. **b** Pulse trains in the time domain. **c** Optical spectrum, and **d** autocorrelation trajectory of the soliton pulse. The pump power is 140 mW

According to the radio frequency spectrum illustrated in Fig. 6a, stable mode-locking was realized at pump power of 140 mW and the laser operated at the repetition frequency of 0.903 MHz with the SNR of 72 dB. The dissipative soliton pulse train is shown in Fig. 6b, with a peak-to-peak spacing of 1.1 μs, which matched the cavity length of 227 m. Figure 6c shows the optical spectrum; the center wavelength of the spectrum was 1064.38 nm, the 3 dB bandwidth was 2 nm, and due to the effective gain bandwidth of the Yb-doped fiber the spectrum had steep side peaks. The influence of gain saturation and nonlinear effects made the peaks in the main spectrum reach the same height. The typical rectangular spectrum characteristics indicate that the laser output pulses was in the form of dissipative soliton pulses [40, 41]. The autocorrelation curve of the pulse train is shown in Fig. 6d, and a Gaussian function was used to fit the autocorrelation curve to obtain a pulse width of 1.28 ps, and by calculation, the time-bandwidth product was 0.658. Compared to that of the transform-limited Gaussian pulse of 0.44, this value indicates the presence of chirp in the pulses.

We observed the pulse sequence and spectrum at a pump power of 270 mW for three hours over three days to investigate the long-term stability of dissipative soliton mode-locking and the BP/SnSe_2_ mixture SA. The pulse sequence and spectrum were always stable during the experimental period, demonstrating the long-term stability of the mode-locking state. There was no damage to the SA material throughout the whole experiment process, proving the robustness and excellent stability of the mixture SA.

## Conclusion

Stable dissipative soliton generation were successfully achieved in an YDFL using the mixture of BP/SnSe_2_-PVA as a SA; the pulse width was 1.28 ps at pump power of 140 mW, the repetition rate was 0.903 MHz, and the center wavelength was 1064.38 nm. The laser achieved the maximum pulse energy of 4.43 nJ at pump power of 140 mW with SNR of 72 dB. The findings demonstrate that the BP/SnSe_2_-PVA mixture SA has outstanding nonlinear saturable absorption characteristics, and broad application prospects in ultrafast lasers.

## Data Availability

The data that support the findings of this study are available from the corresponding author, upon reasonable request.
